# Correlation analysis of the abdominal visceral fat area with the structure and function of the heart and liver in obesity: a prospective magnetic resonance imaging study

**DOI:** 10.1186/s12933-023-01926-0

**Published:** 2023-08-10

**Authors:** Jinquan Bai, Chao Gao, Xiaolu Li, Hong Pan, Shuting Wang, Zhenzhou Shi, Tong Zhang

**Affiliations:** Department of Radiology, The Fourth Affiliated Hospital of Harbin Medical University, Harbin, China

**Keywords:** Obesity, Visceral fat area, Cardiac disease, Nonalcoholic fatty liver disease, Magnetic resonance imaging

## Abstract

**Background:**

The differences in fat deposition sites exhibit varying degrees of systemic inflammatory responses and organ damage, especially in obese individuals with excessive visceral fat. Visceral fat, which is closely related to an increase in mortality rates related to heart and liver diseases. However, few studies have analysed the differences in heart and liver indicators and their correlation among groups based on the abdominal visceral fat area (AVFA).

**Objective:**

Clarifying the differences in and correlations of heart and liver indicators among groups with different severities of AVFA by magnetic resonance imaging (MRI).

**Methods:**

Sixty-nine subjects with obesity were enrolled. The study group consisted of forty-one individuals (AVFA ≥ 150 cm^2^), and the control group consisted of twenty-eight individuals (100 cm^2^ ≤ AVFA < 150 cm^2^). The differences in and correlations between clinical, laboratory, and MRI indicators of the heart and liver between the two groups were analysed.

**Results:**

In the study group, the incidences of type 2 diabetes mellitus (T2DM) and insulin resistance were higher, and liver function indicators were worse. The left ventricular eccentricity ratio (LVER), left ventricular mass (LVM) and global peak wall thickness (GPWT) were higher in the study group than in the control group (*P* = 0.002, *P* = 0.001, *P* = 0.03), and the left ventricle global longitudinal strain (LVGLS) was lower in the study group than in the control group (*P* = 0.016). The pericardiac adipose tissue volume (PATV) and myocardial proton density fat fraction (M-PDFF) were higher in the study group than in the control group (*P* = 0.001, *P* = 0.001). The hepatic proton density fat fraction (H-PDFF) and abdominal subcutaneous fat area (ASFA) were higher in the study group than in the control group (*P* < 0.001, *P* = 0.012). There was a moderate positive correlation (ρ = 0.39–0.59, *P* < 0.001) between the AVFA and LVER, LVM, GPWT, LVGLS, and H-PDFF. There was no difference in right ventricular and most left ventricular systolic and diastolic function between the two groups.

**Conclusion:**

The high AVFA group had a larger LVM, GPWT and PATV, more obvious changes in LVER, impaired left ventricular diastolic function, an increased risk of heart disease, and more severe hepatic fat deposition and liver injury. Therefore, there is a correlation between the amount of visceral adipose tissue and subclinical cardiac changes and liver injury.

**Supplementary Information:**

The online version contains supplementary material available at 10.1186/s12933-023-01926-0.

## Introduction

Individuals with obesity have different degrees of body fat distribution, metabolic characteristics, and related heart and liver damage. Obesity causes fat accumulation in visceral adipose tissue (VAT) and subcutaneous adipose tissue (SAT) and excessive ectopic fat deposition in the liver and myocardium, which is associated with metabolic disorders that lead to various metabolism-related diseases throughout the body [1]. Research has found that the lipolytic activity of VAT is much higher than that of SAT, and the level of lipolysis is affected when insulin function is impaired. This is especially true in adipose tissue, which is the most sensitive tissue to the insulin response. The excessive deposition of VAT also reflects the severity of insulin resistance, and VAT, not SAT, is independently associated with LV remodelling [2, 3]. One possible explanation for this association is that VAT can secrete more cytokines, such as TNF-α, IL-1 and other factors, leading to systemic low-grade inflammation and abnormal cardiac metabolism [4, 5]. In the case of long-term insulin resistance, cardiac tissue is exposed to high levels of fatty acids and blood sugar due to the imbalance between the intake of fatty acids and β-oxidation. The accumulation of lipids in the myocardium and epicardium leads to cardiac steatosis and an increase in epicardium fat volume, and some inflammatory factors are secreted. This leads to myocardial mitochondrial dysfunction, which is the main cause of cardiac lipotoxicity and is related to heart failure (HF) [6–8]. Some studies have shown that all abdominal obesity indices are associated with the risk of cardiovascular (CV) events, highlighting that the Chinese visceral adiposity index (CVAI) might be a valuable abdominal obesity indicator for these events in populations with T2DM [9], and the body shape index (ABSI) is positively associated with all-cause and cardiovascular disease mortality among the general Chinese population with normal BMI. These findings suggest that the ABSI may be an effective tool for central fatness and mortality risk assessment [10]. Multiple research reports have also analysed the correlations between VAT and heart disease and nonalcoholic fatty liver disease (NAFLD) [11, 12].

The liver is an important metabolic organ, and increases in the prevalence and severity of NAFLD are positively correlated with obesity [13]. Metabolic abnormalities, such as the excessive deposition of abdominal visceral adipose tissue (AVAT), are risk factors for the progression of NAFLD [14]. At present, the global prevalence of NAFLD is estimated to be 25–30%, but this prevalence increases to 90% among morbidly patients with obesity with predominantly abdominal obesity [15]. Obesity-driven NAFLD prevalence and the subsequent incidence rate can be considered a major health crisis in the next decade [16]. Patients with the simultaneous progression of simple fatty liver to nonalcoholic steatohepatitis (NASH) and fibrosis can be identified as a special cardiac risk group, as these patients have the highest rates of cardiovascular adverse events and mortality [2].

The metabolic activity of visceral adipose tissue (VAT) is considered a key factor in the occurrence of obesity-related complications [17]. Previous studies have mainly analysed the impact and correlation of VAT on heart and liver diseases without using VAT as a grouping factor to analyse the differences in cardiac and liver magnetic resonance imaging (MRI) indicators. Moreover, ultrasound is often used for the functional evaluation of the heart and liver, but the use of MRI is relatively rare. MRI is a multiparameter, noninvasive examination method that can analyse the structure, function, and movement of the heart, the fat content of myocardial tissue and the volume of pericardiac adipose tissue (including epicardial and paracardiac adipose tissue) [18], and it can quantitatively measure fat in the liver and abdomen with good repeatability and accuracy. Therefore, the purpose of this study is to apply MRI to clarify the differences in cardiac and liver indicators between groups and to identify correlations based on different severities of AVFA as grouping conditions.

## Methods

### Participants

From September 2021 to April 2023, 69 subjects with obesity were recruited from the Weight Loss and Metabolic Surgery Department of the Fourth Affiliated Hospital of Harbin Medical University. Clinical data and laboratory examinations were collected at admission, and cardiac and abdominal scans were performed using a 3.0 T MRI. The visceral fat area was significantly increased (AVFA ≥ 150 cm^2^) in the study group (41 subjects), and the AVFA was slightly increased (100 cm^2^ ≤ AVFA < 150 cm^2^) in the control group (28 subjects). The exclusion criteria included contraindications to MRI, such as large abdominal circumference and claustrophobia, history of heart disease, and history of alcohol or drug use leading to NAFLD. All subjects were not obese due to abnormal hormone levels. The characteristics of the subjects are shown in Table 1.

**Table 1:** Clinical baseline characteristics

Variable	100 cm^2^ < VFA < 150 cm^2^	VFA ≥ 150 cm^2^	*P value*
Number	N = 28	N = 41	
Age, year	32.4 (8.84)	33.7 (11.1)	0.604
Female sex, n (%)	19 (68%)	29 (71%)	0.797
Weight, kg	106 (20.3)	109 (21.4)	0.613
Height, cm	166 (7.15)	166 (6.93)	0.851
Body mass index, kg/m^2^	38.4 (6.27)	38.9 (5.53)	0.76
Body surface area, m^2^	2.18 (0.20)	2.26 (0.28)	0.171
Obesity year	13.5 (9.4)	14.1 (10.2)	0.814
Diabetes mellitus, n (%)	10 (36%)	30 (73%)	0.003
Medication usage, n (%)	2 (20%)	7 (23%)	NA
Dyslipidemia, n (%)	18 (64%)	30 (73%)	0.441
Medication usage, n (%)	3 (17%)	6 (20%)	NA
Steatosis, %	23 (82%)	39 (95%)	0.111
Systolic blood pressure, mmHg	130 (5.4)	132 (9.1)	0.429
Diastolic blood pressure, mmHg	75.6 (8.2)	78.6 (9.1)	0.289
AST, U/l	25.0 (15.4)	33.3 (17.6)	0.043
ALT, U/l	30.9 (22.3)	43.7 (31.6)	0.053
Elevated AST, ALT, n (%)	4 (14%)	18 (44%)	0.017
GGT, U/l	34.9 (15.1)	56.9 (40.2)	0.002
Elevated GGT, n (%)	2 (7%)	14 (34%)	0.010
Platelets, 1000/mm^3^	297 (45.1)	287 (59.6)	0.432
Albumin, g/l	43.3 (3.74)	41.5 (3.64)	0.044
Glucose, mmol/l	5.18 (0.77)	7.04 (3.17)	0.001
Glycated hemoglobin, %	5.64 (0.75)	6.94 (1.92)	< 0.001
Total cholesterol, mmol/l	4.91 (0.85)	5.45 (0.90)	0.013
Triglycerides, mmol/l	1.33 (0.54)	3.04 (4.65)	0.025
High-density lipoprotein, mmol/l	1.19 (0.16)	1.06 (0.23)	0.009
Low-density lipoprotein, mmol/l	2.82 (0.61)	3.05 (0.81)	0.186
HOMA-IR	5.77 (2.58)	8.63 (4.55)	0.001
NFS	− 2.48 (1.46)	− 1.91 (1.48)	0.123

Data are presented as the mean (SD) or median (interquartile range)

*AST* aspartate aminotransferase, *ALT* alanine aminotransferase, *GGT* gamma-glutamyl transferase, *HOMA-IR* homeostasis model assessment- insulin resistance, *NFS* NAFLD fibrosis score, *NAFLD* nonalcoholic fatty liver disease

**Fig. 1:**
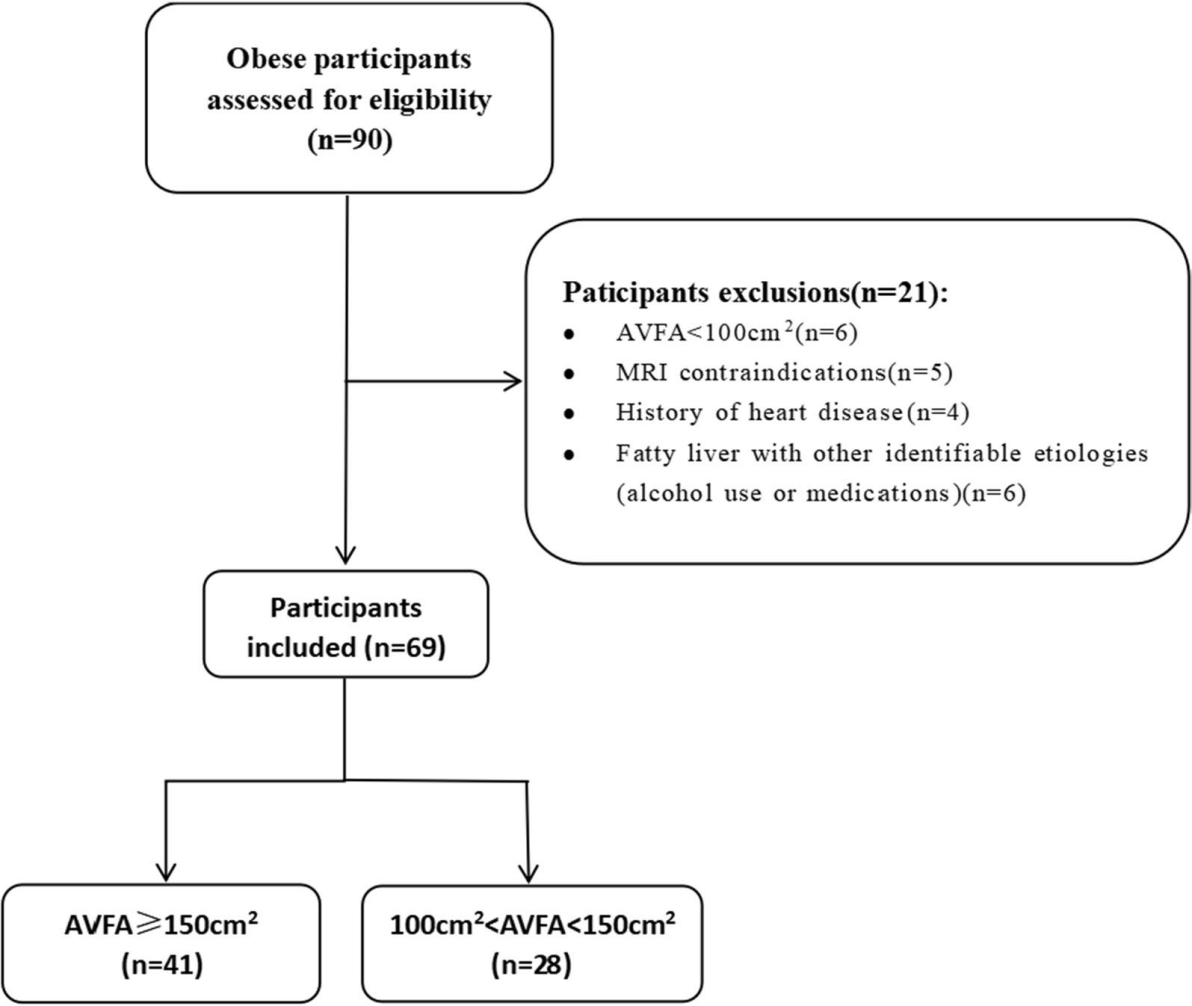
Flowchart showing the inclusion and exclusion criteria for the study population

Before collecting clinical samples, all patients provided written informed consent. This study was conducted in accordance with the standards established by the Declaration of Helsinki. The study protocol was approved by the Ethics Committee of the Fourth Affiliated Hospital of Harbin Medical University. Fig. 1 illustrates the selection and exclusion of subjects.

### HOMA-IR and NFS

The homeostasis model assessment- insulin resistance index (HOMA-IR index) was calculated by the formula HOMA-IR = [glucose (mmol/l) × insulin (µU/ml)]/22.5 [19].

Basic information, such as age and body mass index (BMI), and blood and biochemical indicators were collected. Nonalcoholic fatty liver disease (NAFLD) is defined as the presence of ≥ 5% hepatic steatosis [20], which was assessed quantitatively based on MRI (mDixon-quant sequence). Among subjects with NAFLD, the presence of advanced liver fibrosis was determined by the NAFLD fibrosis score (NFS): − 1.675 + 0.037 × age (year) + 0.094 × BMI (kg/m^2^) + 1.13 × (impaired fasting glucose or diabetes mellitus) + 0.99 × AST/ALT − 0.013 × platelet (10^−9^/l) − 0.66 × albumin (g/dl) [21].

### Cardiac MRI

**Fig. 2:**
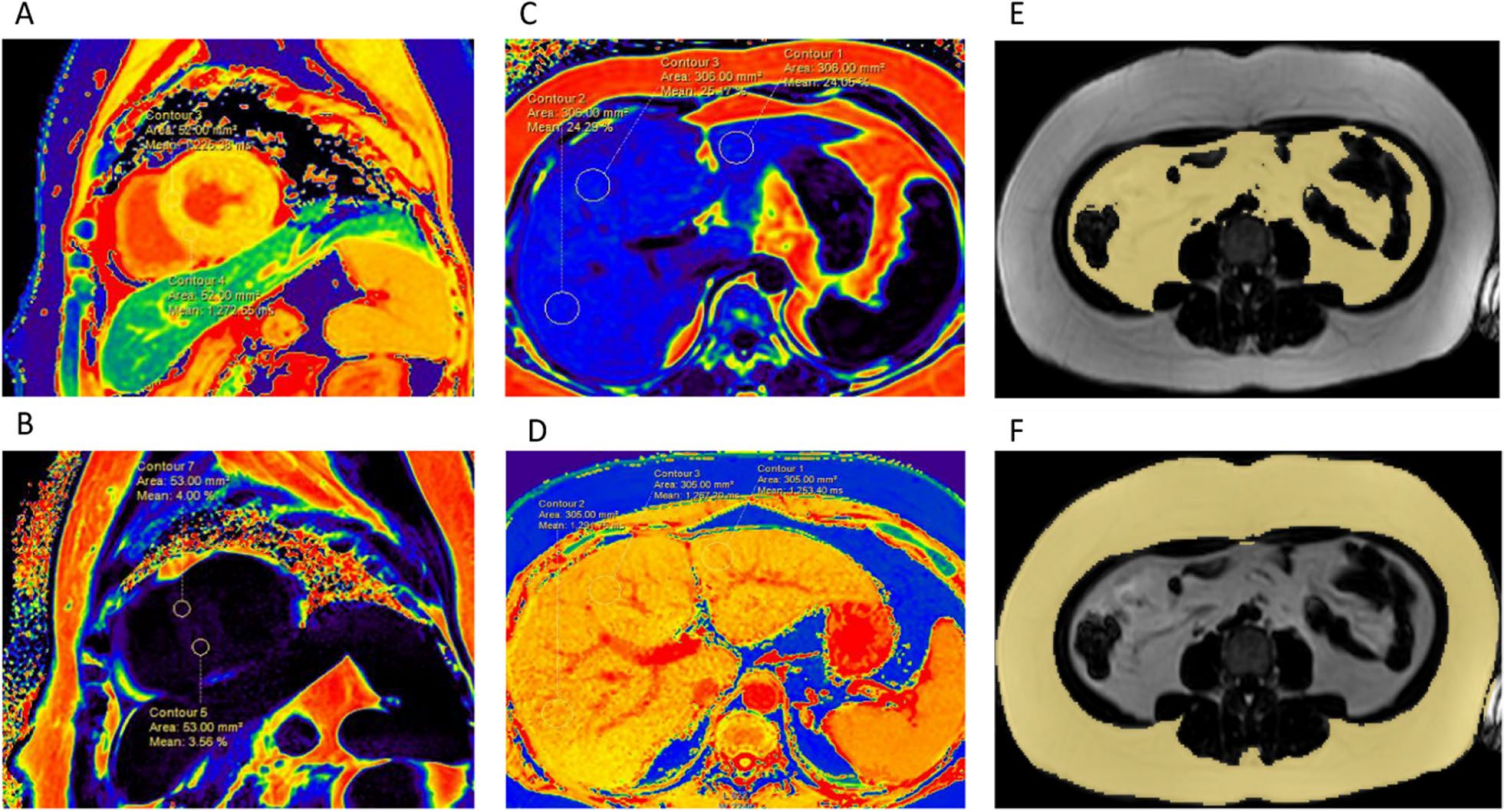
MRI diagram. **A** Myocardial native T1 mapping; **B** myocardial proton density fat fraction (M-PDFF); **C** hepatic proton density fat fraction (H-PDFF); **D** hepatic native T1 mapping; **E** abdominal visceral fat area (AVFA); **F** abdominal subcutaneous fat area (ASFA)

A wide-bore 3 T magnetic resonance scanner (Ingenia, Philips, Holland) with a diameter of 70 cm was used, and a 32-channel cardiac coil (Philips) was used with the participants in a supine position and electrocardiogram (ECG) gating. The examination sequences included the left ventricular long axis, four chambers, and left ventricular short axis cardiac cine (field of view (FOV) = 300 × 320 mm^2^, repetition time (TR)/echo time (TE) = 3.5/1.76 ms, flip angle 45°, slice thickness 8 mm, 0 mm gap, 10 slices, 30 phases). Native T1 mapping [modified Look-Locker (3(3)3(3)5 MOLLI], FOV 320 × 320 mm^2^, TR/TE 2.3/1.08 ms, slice thickness 8 mm, flip angle 20°, matrix 160 × 160) and multiecho (six-echo) Water-fat mDixon-quant MRI (acquisition using compressed sensing technology, FOV = 300 × 320 mm^2^, TR/TE1/delta TE = 8.1/1.47/1.0 ms, slice thickness 8 mm, flip angle 3°, matrix 216 × 216) were performed. CVI42 v5.11.3 (Circle Cardiovascular Imaging, Calgary, Canada) was used to analyse the structure and function of the left and right ventricles (artificial intelligence automatically delineates the endocardium and epicardium). The native T1 value (avoiding signal pollution points), pericardiac adipose tissue volume (PATV) (cardiac short axis cine sequence bottom to apex manual delineation plus threshold setting measurement), and myocardial strain was measured using myocardial feature tracking technology to measure left and right ventricular global radial strain (GRS), global circumferential strain (GCS), and global longitudinal strain (GLS) [22]. The M-PDFF was measured by a Philips workstation in the proton density fat fraction (PDFF) map at the short axis plane of the middle segment of the left ventricle, and the regions of interest (ROI) area was 50 ± 5 mm^2^ (Fig. 2A, B). The left ventricular eccentricity ratio (left ventricular mass/end diastolic blood volume) is an indicator of concentric remodelling.

### Abdomen MRI

All subjects fasted for at least 4 h before examination. The scanning range was from the upper margin of the liver to the level of the anterior superior iliac crest. An axial three-dimensional multiecho (six-echo) mDixon sequence was acquired for hepatic proton density fat fraction (H-PDFF) measurement within 16 s during one breath hold (FOV 500 × 450 mm^2^, TR/TE1/delta TE 5.5/0.93/0.7 ms, slice thickness 3.5 mm, flip angle 4°, matrix size 200 × 189). Native T1 mapping was acquired by using the modified Look-Locker inversion recovery (MOLLI) (FOV 500 × 450 mm^2^, TR/TE 3.2/1.32 ms, inversion time (TI) 139 ms, slice thickness 5 mm, flip angle 8°, matrix 192 × 150). Three slices were obtained for each patient. Free-breathing and respiratory triggered diffusion-weighted imaging (DWI) was performed using b values between 0 and 1000 s/mm^2^, and then the apparent diffusion coefficient (ADC) value of the liver was measured, which is used to describe the speed and range of molecular diffusion motion in different directions in the DWI sequence. All sequences were manually delineated into three ROIs (on the level of the right branch of the portal vein entering the liver, ROIs with an area of 300 ± 10 mm^2^ were placed in the left lobe, the right anterior lobe and the right posterior lobe of the liver, and the average value was measured, avoiding blood vessels and bile ducts) (Fig. 2C, D). The abdominal visceral fat area and subcutaneous fat area were sketched by manual sketching and the threshold adjustment semiautomatic method in CVI42 (v5.11.3) and measured horizontally at the lower edge of the third lumbar vertebra [23] (Fig. 2E, F).

### Statistical analysis

Statistical analysis was conducted using R software (version 4.2.2) and SPSS25 (IBM, NY, US). A normal analysis of the data was performed using the Shapiro–Wilk test. Normally distributed variables are expressed as the mean ± standard deviation (SD). For variables with nonnormal distributions, data are expressed as the median and interquartile range. Categorical data are presented as the number and percentage of cases in each category. Analysing the correlation between the AVFA and cardiac and whole-body MRI parameters is a two-step process. First, the difference analysis of clinical and MRI results was conducted for the subjects in the study group and the control group. Student's t test was used for normally distributed variables, and the Mann‒Whitney U test was used for nonnormally distributed variables. The chi-square test was used for categorical variables. Pearson or Spearman correlation analysis was performed for all MRI examination results, HOMA-IR, and NFS. Multivariate analysis was performed for MRI indicators that were correlated with the AVFA using five multiple linear regression models to observe their corrected correlation with AVFA. The models were as follows: Model 1: single correlation with AVFA; Model 2: Model 1 + covariates age, sex, BMI, and years of obesity; Model 3: Model 2 + blood pressure, blood glucose, triglycerides, total cholesterol, high-density lipoprotein cholesterol, low-density lipoprotein cholesterol, AST, ALT, GGT, albumin, glycosylated haemoglobin, HOMA-IR, and NFS; Model 4: Model 3 + body MRI (hepatic proton density fat fraction, abdominal subcutaneous fat area, the values of native T1 and the apparent diffusion coefficient of liver); Model 5: Model 4 + cardiac MRI (left ventricular eccentricity ratio, left ventricular mass, global myocardial wall thickness, left ventricle global longitudinal strain, pericardiac adipose tissue volume, myocardial proton density fat fraction). The collinearity was analysed by paired calculation between covariates and observations of variance inflation factor (VIF) values and tolerance values. A *P* < 0.05 (two-tailed) was considered statistically significant.

## Results

The average age of the 69 participants who were finally enrolled was 33.5 years. The visceral fat area was significantly increased (AVFA ≥ 150 cm^2^) in the study group (41 subjects), and the AVFA was slightly increased (100 cm^2^ ≤ AVFA < 150 cm^2^) in the control group (28 subjects). The proportion of women in both groups was approximately 70%. The average weight was 108 kg (75–154 kg), and the average duration of an obesity status (BMI > 25 kg/cm^2^) was approximately 14 years. There was no significant difference in age, sex, BMI, heart rate, or blood pressure between the two groups. Compared with those of the control group, the glycosylated haemoglobin, HOMA-IR, total cholesterol, low-density lipoprotein cholesterol and triglyceride levels of the subjects in the study group were increased, and the high-density lipoprotein cholesterol level was decreased (Table 1). The laboratory indicators of liver injury were higher, the proportion of patients with type 2 diabetes was higher, and insulin resistance was more serious in the subjects in the study group than in those in the control group.

Compared with the control group, the study group had a higher left ventricular eccentricity ratio (LVER) (0.80 ± 0.13 vs. 0.72 ± 0.11 g/ml, *P* = 0.002), higher left ventricular global peak wall thickness (GPWT) (11.8 [9.27, 13.4] vs. 10.3 [9.27, 11.99] g/ml, *P* = 0.03), and higher LVM (114 ± 29.6 vs. 93.3 ± 18.7 g, *P* = 0.001), but there was no significant difference between the two groups after correcting for body surface area (BSA) (48.9 ± 12.0 vs. 44.3 ± 7.55 g, *P* = 0.053). The left ventricle global longitudinal strain (LVGLS) of the study group was lower than that of the control group (− 13.31% ± − 4.04% vs. − 15.22% ± − 2.36%, *P* = 0.016). The pericardial adipose tissue volume (PATV) of the study group was higher than that of the control group (116 ± 41.6 vs. 78.7 ± 20.6 ml, *P* < 0.001). The myocardial proton density fat fraction (M-PDFF) and hepatic proton density fat fraction (H-PDFF) of the study group were higher than those of the control group (2.93 ± 0.78 vs. 2.30 ± 0.71, *P* = 0.001; 20.6% ± 7.81% vs. 12.7% ± 8.49%, *P* < 0.001), and the abdominal subcutaneous fat area (ASFA) of the study group was higher than that of the control group (293 ± 84.4 vs. 237 ± 90.4 ml, *P* = 0.012) (Table 2).

**Table 2:** MRI measurement results of the heart and abdomen

Variable	100 cm^2^ < AVFA < 150 cm^2^	AVFA ≥ 150 cm^2^	*P value*
Number	N = 28	N = 41	
LVEDV, ml/m^2^	132 (24.8)	143 (33.8)	0.127
LVEDV/BSA, ml/m^2^	60.7 (11.0)	63.1 (13.2)	0.412
LVESV, ml	51.2 (12.9)	55.9 (17.0)	0.198
LVESV/BSA, ml/m^2^	23.5 (6.05)	24.7 (6.63)	0.41
LVSV, ml	80.3 (18.4)	86.1 (25.2)	0.277
LVSV/BSA, ml/m^2^	35.7 (8.45)	38.1 (10.8)	0.309
LVEF, %	61.0 (7.44)	60.0 (8.63)	0.607
LVEF/BSA, ml/m^2^	28.1 (4.04)	26.7 (5.09)	0.2
LVCO, 1/min	6.14 (1.28)	6.32 (1.71)	0.633
LVCI, 1/min/m^2^	2.78 (0.48)	2.77 (0.62)	0.951
LVER(g/ml)	0.72 (0.11)	0.80 (0.13)	0.015
LVM, g	93.3 (18.7)	114 (29.6)	0.001
LVM/BSA, ml/m^2^	44.3 (7.55)	48.9 (12.0)	0.053
GPWT, mm	10.3 (9.27, 11.99)	11.8 (9.27, 13.4)	0.03
HR, 1/min	78.2 (11.2)	76.0 (14.0)	0.468
LVGRS, %	30.3 (5.95)	30.8 (7.67)	0.756
LVGCS, %	− 17.97 (2.13)	− 17.78 (3.01)	0.766
LVGLS, %	− 15.22 (2.36)	− 13.31 (4.04)	0.016
Myocardial T1 values, ms	1262 (47.1)	1253 (46.2)	0.458
PATV, ml	78.7 (20.6)	116 (41.6)	< 0.001
M-PDFF, %	2.30 (0.71)	2.93 (0.78)	0.001
AVFA, cm^2^	117.5 (105.2, 128.5)	212 (185.5, 255.5)	< 0.001
ASFA, cm^2^	237 (90.4)	293 (84.4)	0.012
Hepatic T1 values, ms	1040 (933.84, 1098.27)	1073 (928.44, 1191.63)	0.344
H-PDFF, %	12.7 (8.49)	20.6 (7.81)	< 0.001
Hepatic ADC, 10^−3^mm^2^/s	1.14 (0.43)	0.95 (0.43)	0.076

Data are presented as the mean (SD) or median (interquartile range)

*LV* left ventricle, *EDV* end diastolic volume, *BSA* body surface area, *ESV* end systolic volume, *SV* stroke volume, *EF* ejection fraction, *CO* cardiac output (the amount of blood pumped by the heart per minute), *CI* cardiac index (CO/BSA), *ER* eccentricity ratio, *LVM* left ventricular mass, *GPWT* global peak wall thickness, *HR* heart rate, *GRS* global radial strain, *GCS* global circumferential strain, *GLS* global longitudinal strain, *PATV* pericardiac adipose tissue volume, *M-PDFF* myocardial proton density fat fraction, *AVFA* abdominal visceral fat area, *ASFA* abdominal subcutaneous fat area, *H-PDFF* hepatic proton density fat fraction, *ADC* apparent diffusion coefficient

**Fig. 3:**
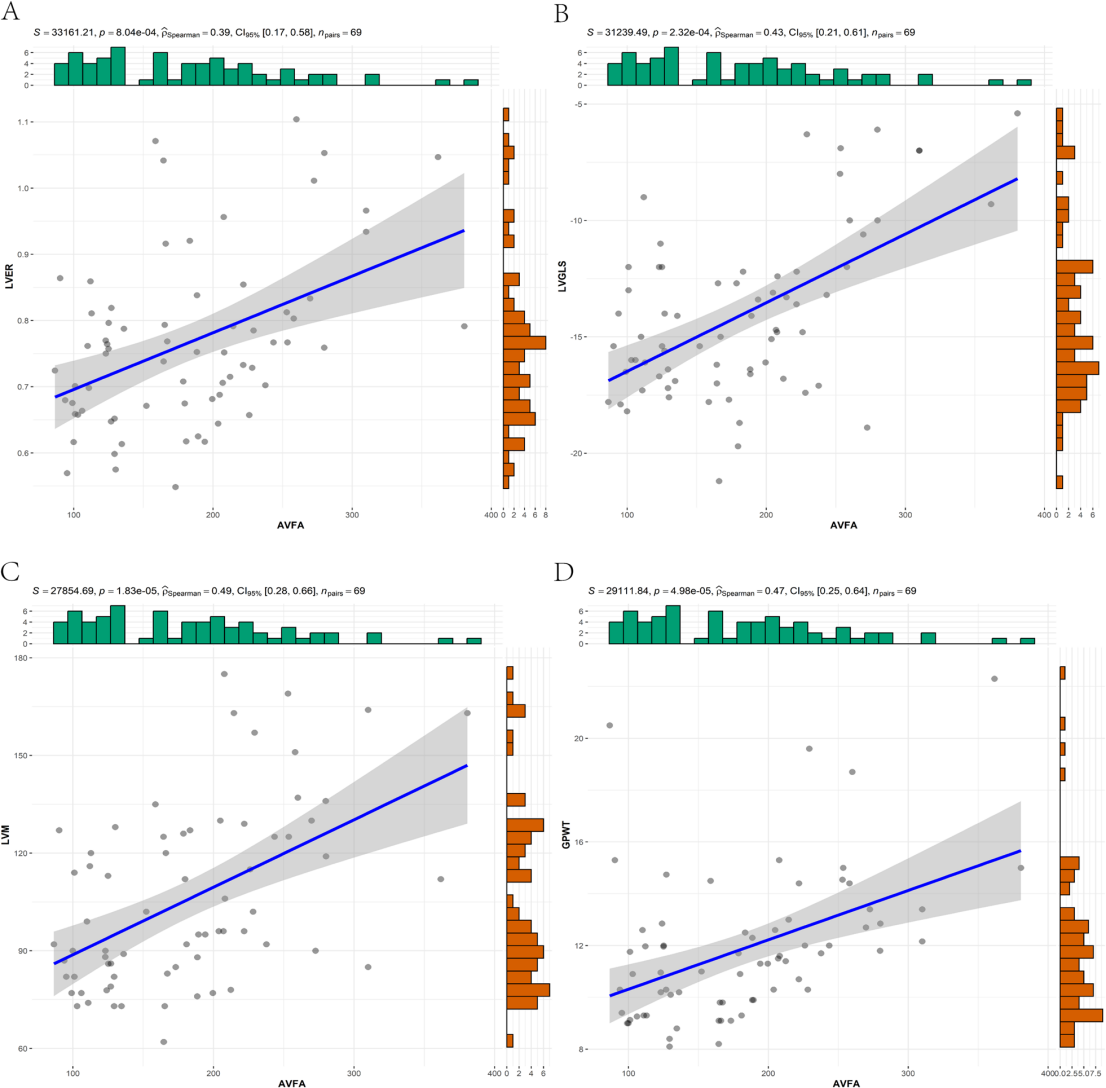
Pearson correlation analysis between abdominal visceral fat area and cardiac MRI results. **A**-**D** shows the correlation between AVFA and LVER, LVGLS, LVM and GPWT respectively. The statistics at the top of the graph include the P value of indexation, the ρ value of correlation, the 95% confidence interval and the number of statistical samples. *LVER* left ventricle eccentricity ratio, *LVM* left ventricular mass, *LVGLS* left ventricular global radial strain, *GPWT* global peak wall thickness

**Fig. 4:**
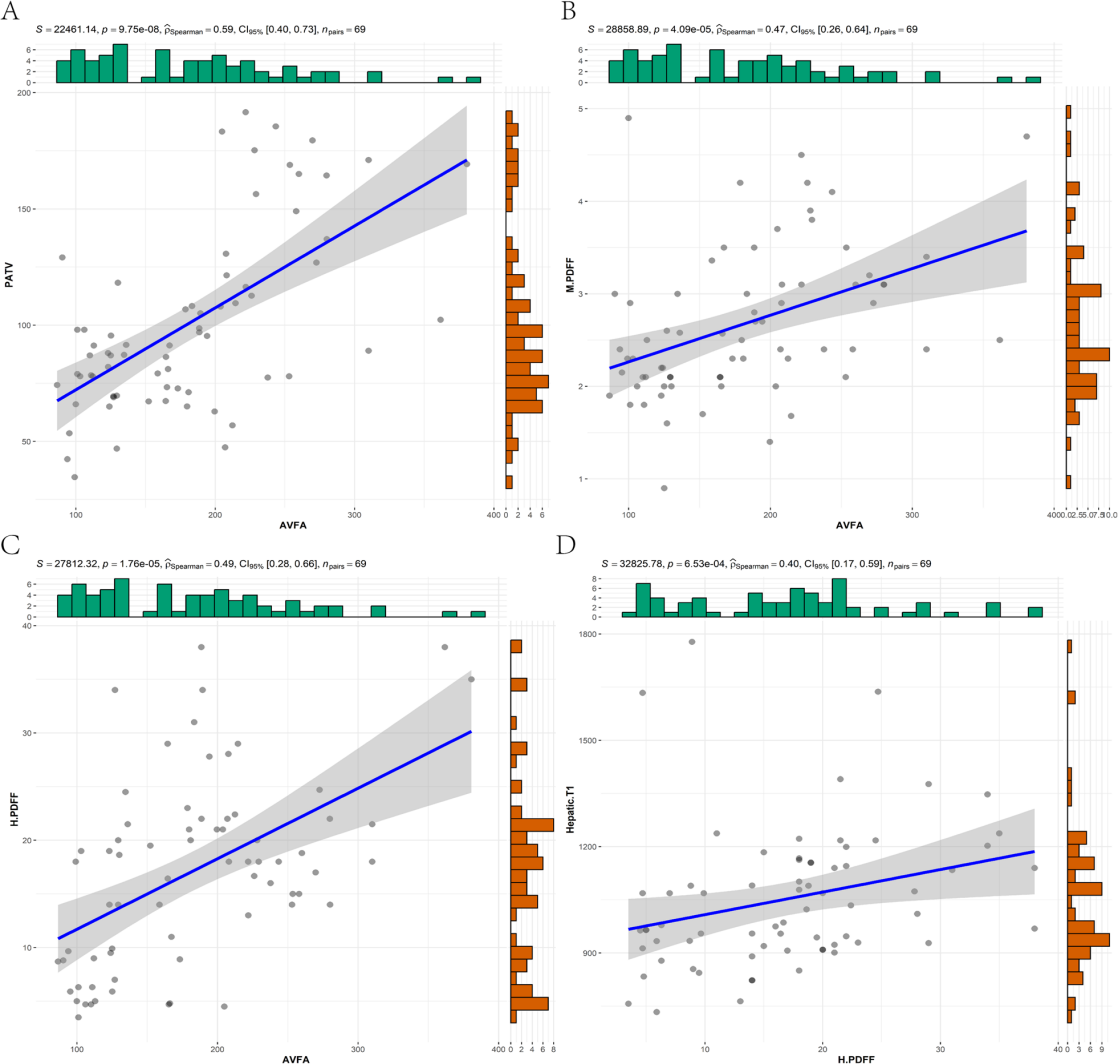
Pearson correlation analysis between cardiac and abdominal MRI results. **A**-**C** shows the correlation between AVFA and PATV, M-PDFF, H-PDFF respectively, D shows the correlation between H-PDFF and hepatic T1 values. The statistics at the top of the graph include the P value of indexation, the ρ value of correlation, the 95% confidence interval and the number of statistical samples. *AVFA* abdominal visceral fat area, *PATV* pericardiac adipose tissue volume, *M-PDFF* myocardial proton density fat fraction, *H-PDFF* hepatic proton density fat fraction

The AVFA had a moderate positive correlation with LVER, LVM, LVGLS, PATV, and M-PDFF (ρ = 0.39–0.59, *P* < 0.001) (Figs. 3, 4). After adjusting for clinical and laboratory indicators in Models 2–4, the correlation between the AVFA and cardiac MRI indicators slightly weakened. After adding other cardiac MRI indicators to Model 5, the AVFA still had a slight correlation with LVER and LVGLS, but it was not correlated with left ventricular mass (LVM), PATV and M-PDFF. There was a moderate positive correlation between the AVFA and H-PDFF (ρ = 0.49, *P* < 0.001) (Fig. 4), and the correlation slightly weakened after adjustment for all variables but was still statistically significant. There is a slight correlation between the AVFA and ASFA (ρ = 0.289, *P* < 0.016), which still existed after correction for Models 2 and 3. However, the correlation disappeared after adding MRI indicators in Models 4 and 5 (Table 3). We found that BSA was correlated with LVER (r = 0.29, P < 0.05) but not with LVM (Additional file 1). There was no difference in systolic and diastolic function between the right ventricle and most left ventricles between the two groups.

**Table 3:** Associations between AVFA and cardiac and abdominal MRI results by multivariable linear regression

	Model 1 (β[SE], *P*, [95%CI])	Model 2 (β[SE], *P*, [95%CI])	Model 3 (β[SE], *P*, [95%CI])	Model 4 (β[SE],*P*, [95%CI])	Model 5 (β[SE],*P*, [95%CI])
LVER	0.453(0.109), *P* < 0.001, (0.235, 0.670)	0.420(0.106), *P* < 0.001, (0.208, 0.632)	0.496(0.149), *P* = 0.002, (0.197, 0.796)	0.525(0.163), *P* = 0.002, (0.197, 0.853)	0.398(0.193), *P* = 0.050, (0.000, 0.797)
LVM	0.514(0.105), P < 0.001, (0.305, 0.723)	0.512(0.107), *P* < 0.001, (0.298, 0.726)	0.435(0.128), *P* = 0.001, (0.178, 0.692)	0.355(0.151), *P* = 0.023, (0.051, 0.658)	0.151(0.187), *P* = 0.423, (− 0.225, 0.528)
LVGLS	0.561(0.101), P < 0.001, (0.359, 0.763)	0.541(0.102), *P* < 0.001, (0.336, 0.746)	0.460(0.127), *P* = 0.001, (0.205, 0.715)	0.482(0.153), *P* = 0.003, (0.173, 0.789)	0.391(0.193), *P* = 0.049, (0.002, 0.780)
PATV	0.612(0.097), P < 0.001, (0.419, 0.805)	0.610(0.097), *P* < 0.001, (0.415, 0.805)	0.511(0.125), *P* < 0.001, (0.259, 0.763)	0.530(0.141), *P* < 0.001, (0.246, 0.813)	0.176(0.160), *P* = 0.277, (− 0.147, 0.499)
M-PDFF	0.430(0.110), P < 0.001, (0.210, 0.650)	0.432(0.113), *P* < 0.001, (0.206, 0.659)	0.550(0.151), *P* = 0.001, (0.247, 0.853)	0.565(0.162), *P* = 0.001, (0.239, 0.892)	0.320(0.186), *P* = 0.092, (− 0.540, 0.695)
H-PDFF	0.504(0.106), P < 0.001, (0.293, 0.715)	0.500(0.109), *P* < 0.001, (0.282, 0.718)	0.470(0.132), *P* = 0.001, (0.205, 0.736)	0.417(0.135), *P* = 0.003, (0.145, 0.689)	0.465(0.181), *P* = 0.014, (0.099, 0.830)
ASFA	0.289(0.117), P = 0.016, (0.056, 0.523)	0.305(0.118), *P* = 0.012, (0.069, 0.541)	0.383(0.159), *P* = 0.020, (0.063, 0.703)	0.304(0.180), *P* = 0.098, (− 0.058, 0.665)	0.218(0.224), *P* = 0.336, (− 0.234, 0.669)

*AVFA* abdominal visceral fat area, *LVER* left ventricle eccentricity ratio, *LVM* left ventricular mass, *LVGLS* left ventricular global radial strain, *PATV* pericardiac adipose tissue volume, *M-PDFF* myocardial proton density fat fraction, *H-PDFF* hepatic proton density fat fraction, *ASFA* abdominal subcutaneous fat area

## Discussion

This study included 69 subjects with obesity who were not obese due to abnormal hormone levels. The correlations between abdominal visceral adipose tissue (AVAT) and the heart and liver were assessed. AVAT refers to the fat surrounding abdominal viscera, which is more highly related to lipid decomposition and inflammatory cytokine secretion than subcutaneous adipose tissue. There was a significant difference in heart and liver function between the study group (41 subjects) and the control group (28 subjects), and the study group exhibited subclinical changes in heart function, significant liver function damage and more severe fatty liver. Since all subjects were obese, there was no significant difference in BMI between the two groups, and there was no significant difference in age, sex, blood pressure, or heart rate between the two groups. Compared with that in the control group, the proportion of type 2 diabetes mellitus (T2DM) in the study group was significantly higher, and the hepatic proton density fat fraction (H-PDFF) was also higher than that in the control group. Previous studies have shown that T2DM and nonalcoholic fatty liver disease (NAFLD) in subjects with normal weight will affect cardiac function, resulting in concentric remodelling and impaired diastolic function of the left ventricle [24, 25]. However, in individuals with obesity, we did not find any difference in cardiac function between the T2DM and simple fatty liver groups, which is consistent with the research conclusions of VanHirose K and Styczynski G et al. [26, 27]. This indicates that early metabolic abnormalities in patients with obesity have a limited impact on cardiac function. Additionally, the systemic inflammatory response manifested by a further increase in AVAT may have a significant impact on cardiac function, which is an important influencing factor of changes in cardiac function [11].

The latest research of Qu Y et al. [28] found that VAT is related to left ventricular myocardial strain. Our data show that the left ventricle global longitudinal strain (LVGLS) of the subjects in the study group was weakened. Patients with obesity need to increase myocardial torque to ensure a larger cardiac output so they can increase force and pressure to maintain systolic function [29]. In the study group, left ventricular mass (LVM) and global peak wall thickness (GPWT) were increased, and concentric remodelling changes occurred. Excessive VAT deposition leads to increased secretion of adipocytokines, such as TNF-α, IL-1, adiponectin, and leptin, which can cause heart and liver damage. In addition to the role of VAT itself, there were more patients with T2DM in the study group. High glucose levels increase oxidative stress, promote myocardial cell injury and hypertrophy, promote fibrosis under the endocardium, and weaken myocardial strain. The deposition of advanced glycation end products, fibrosis, and an increase in the resting tension of myocardial cells will also lead to left ventricular diastolic stiffness [29, 30]. The visceral adiposity index (VAI) is associated with an increased risk of T2DM [31], in addition to the heart and liver, the VAI and Chinese visceral adiposity index (CVAI) are independently associated with the development of nephropathy but not retinopathy in Chinese adults with T2DM [32]. However, subjects in the study group had higher NFS scores. More severe fatty liver disease can lead to nonalcoholic steatohepatitis and liver fibrosis. This increases the release of proinflammatory cytokines, such as tumour necrosis factor-α, interleukin-6, monocyte chemoattractant protein-1, and other factors, and coagulation-promoting factors and adhesion molecules, which are also associated with myocardial oxidative stress and endothelial dysfunction [33–35], leading to a vicious cycle.

We also found that the abdominal visceral fat area (AVFA) was moderately positively correlated with pericardiac adipose tissue volume (PATV) and myocardial proton density fat fraction (M-PDFF), and the AVFA was higher in the study group than in the control group. NGonz á lez et al. [36] found that VAT may also be associated with epicardial fat accumulation by secreting lipids, fat factors, and proinflammatory and oxidative factors from adipocytes. Thus, VAT is a risk factor for different forms of heart disease and heart failure, mainly in obese subjects. Excessive visceral fat can lead to lipid deposition in nonadipose tissue, especially in the liver and heart [37]. Excessive fat deposition in the myocardium directly affects myocardial function through lipotoxicity [38]. We found that there was a correlation between BSA and LVER, probably because BSA had a certain influence on LVER. There was no correlation between BSA and LVM, which may be because both groups of subjects are obese. Additionally, there is no difference in BMI, so a correlation between these parameters in the general population may exist. In addition, due to the physical limitations of the epicardium, the surrounding ectopic fat pool may mechanically hinder diastolic filling. Thus, pericardial fat volume is associated with left ventricular diastolic dysfunction, independent of traditional risk factors and BMI [39]. Therefore, the release of mechanical, lipotoxic and various proinflammatory mediators may play an important role in cardiac pathophysiology and accelerate this vicious cycle in obese people (Additional file 1).

As expected, the laboratory markers of liver injury in the study group were significantly higher than those in the control group. The degree of hepatic steatosis was significantly higher in the study group than in the control group, and the correlation between the AVFA and H-PDFF was significantly higher than that between the abdominal subcutaneous fat area (ASFA) and H-PDFF. The native T1 values of the liver also increased with the degree of hepatic steatosis (Fig. 4D), which was contrary to the expected negative correlation between the H-PDFF and the native T1 values. However, Ahn JH et al. [40] also demonstrated that the liver fat content was positively correlated with native T1 values when the liver fat content was less than 30%. Although there was no significant difference in the hepatic apparent diffusion coefficient value and NFS between the two groups, the indicators of the study group still showed more obvious liver injury than the control group. These findings indicate that the liver diffusion function of the study group was weakened, which may be related to the decrease in liver cell diffusion function caused by hepatic steatosis and liver fibrosis. This was also demonstrated by Papalavrentios L et al. [41].

In summary, through the analysis of the differences between two groups of subjects with severe obesity and the correlation analysis of different indicators, we found that the study group exhibited impaired liver function and subclinical cardiac function changes, and the AVFA was correlated with cardiac and hepatic MRI results to varying degrees. These findings indicate that excessive AVAT deposition is not only an important factor leading to liver injury but also an important factor leading to these subclinical cardiac changes. Moreover, after correcting for other influencing factors, the AVFA was still significantly related to heart and liver function. Thus, VAT is an important progressive indicator among many factors affecting heart and liver function in people with obesity, which deserves more clinical attention.

## Limitations

Previous studies mostly used echocardiography to evaluate heart and liver function. Echocardiography is a routine examination before bariatric surgery, and it is simple and easy. We chose MRI as the examination method, and subject compliance was poor. The collection of experimental cases is time consuming, and the number of cases of severe obesity in a single centre is still small. Thus, the number of cases is limited. Larger samples from multiple centres are needed to verify the conclusions of this study. Although this study is a prospective study, it is a cross-sectional study. We are currently following up to review the weight loss of these patients with obesity to observe whether the heart and liver structure and function will change after the decrease in AVAT.

## Conclusion

In patients with severe obesity, a significant increase in the AVFA is characterized by changes in LVER. Additionally, it may damage left ventricular diastolic function and increase the risk of heart disease by increasing the PATV. Moreover, the H-PDFF is higher, and liver function is significantly impaired. Therefore, there is a correlation between the amount of visceral adipose tissue and subclinical cardiac changes and liver injury.

### Supplementary Information

**Additional file 1. Figure S1. **Pearson correlation analysis of body surface area with left ventricular eccentricity ratio and left ventricular mass.

## Data Availability

The datasets generated and analyzed during the current study are available from the corresponding authors on reasonable request.

## Abbreviations

ABSI: A body shape index
ALT: Alanine aminotransferase
AST: Aspartate aminotransferase
ASFA: Abdominal subcutaneous fat area
AVFA: Abdominal visceral fat area
BMI: Body mass index
BSA: Body surface area
CVAI: Chinese visceral adiposity index
CV: Cardiovascular
FOV: Field of view
GCS: Global circumferential strain
GLS: Global longitudinal strain
GRS: Global radial strain
GPWT: Global peak wall thickness
GGT: Gamma-glutamyl transferase
HOMA-IR: Homeostasis model assessment of insulin resistance
HF: Heart failure
LVER: Left ventricular eccentricity ratio
LVM: Left ventricular mass
MRI: Magnetic resonance imaging
NAFLD: Nonalcoholic fatty liver disease
NASH: Nonalcoholic steatohepatitis
NFS: NAFLD fibrosis score
PATV: Pericardiac adipose tissue volume
PDFF: Proton density fat fraction
SAT: Subcutaneous adipose tissue
T2DM: Type 2 diabetes mellitus
VAT: Visceral adipose tissue
