# Supplementary figures and images for: Correlation analysis of the abdominal visceral fat area with the structure and function of the heart and liver in obesity: a prospective magnetic resonance imaging study
Source: Cardiovasc Diabetol. 2023 Aug 10;22:206. doi: 10.1186/s12933-023-01926-0 (PMC10416373; doi:10.1186/s12933-023-01926-0)

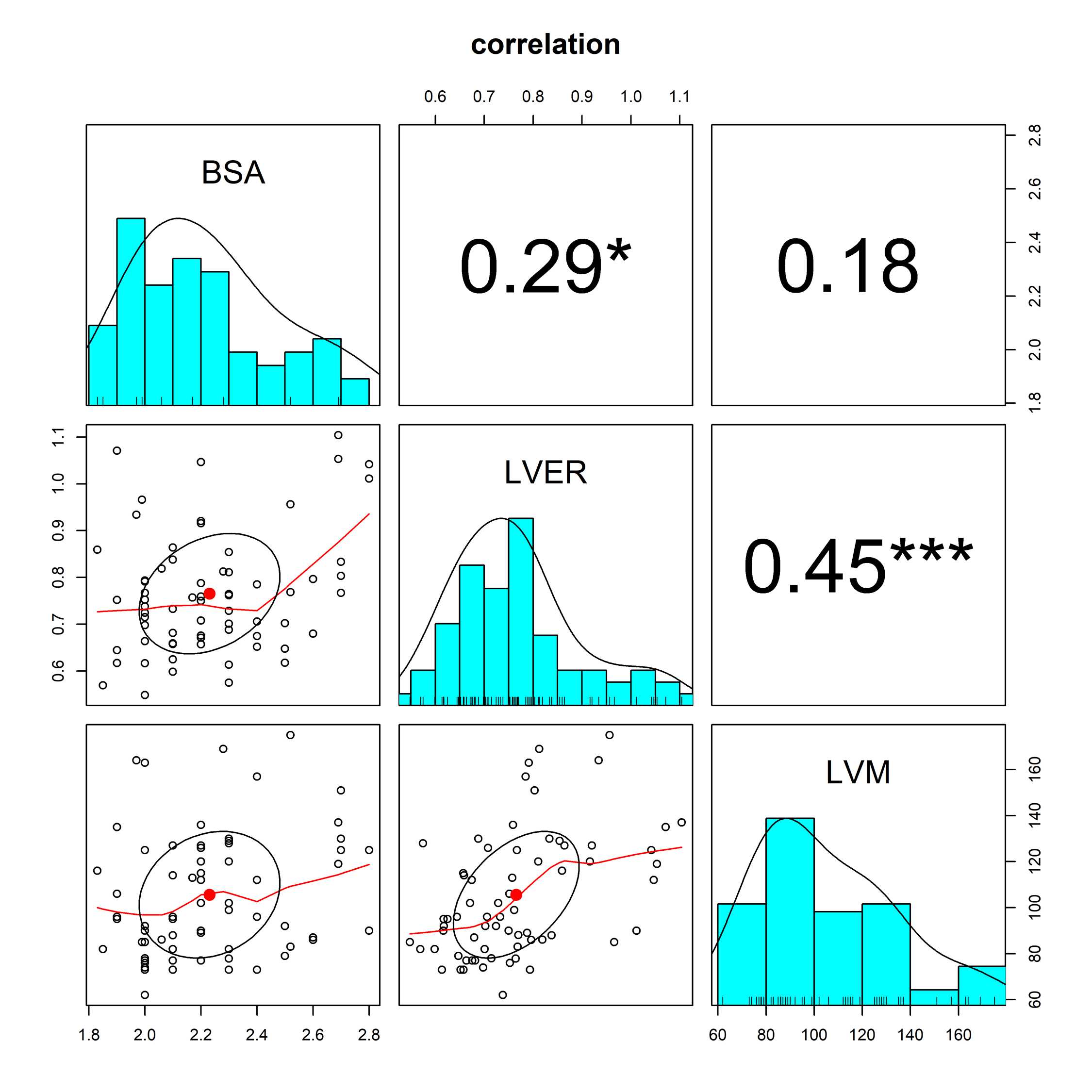

Supplement: Supplementary file 1 — Additional file 1. Figure S1. Pearson correlation analysis of body surface area with left ventricular eccentricity ratio and left ventricular mass. [file 12933_2023_1926_MOESM1_ESM.tif]
